# Application of Genomic SSR Locus Polymorphisms on the Identification and Classification of Chrysanthemum Cultivars in China

**DOI:** 10.1371/journal.pone.0104856

**Published:** 2014-08-22

**Authors:** Yuan Zhang, Silan Dai, Yan Hong, Xuebin Song

**Affiliations:** College of Landscape Architecture, Beijing Forestry University, Beijing, China; USDA-ARS-SRRC, United States of America

## Abstract

The Chinese traditional chrysanthemum is a notable group of chrysanthemums (*Chrysanthemum*×*morifolium* Ramat.) in which the phenotypic characteristics richly vary. At present, there is a serious controversy regarding homonyms and synonyms within this group. Moreover, the current international chrysanthemum classification systems are not comprehensive enough to be used on Chinese traditional chrysanthemums. Thus, we first identified a broad collection of 480 Chinese traditional chrysanthemum cultivars using the unique DNA fingerprints and molecular identities that were established by 20 simple sequence repeat markers. Five loci, which distinguished all of the selected cultivars, were identified as the core loci to establish unique fingerprints and molecular identities with 19 denary digits for each cultivar. A cluster analysis based on Nei's genetic distance indicated that the selected cultivars were clustered according to their horticultural classification. Population structure analysis was subsequently performed with K values ranging from 2 to 14, and the most likely estimate for the population structure was ten subpopulations, which was nearly consistent with the clustering result. Principal component analysis was further performed to verify the classification results. On the basis of the Q-matrices of K = 10, a total of 19 traits were found to be associated with 42 markers. Taken together, these results can serve as starting points for the identification and classification of chrysanthemums based on the polymorphism of microsatellite markers, which is beneficial to promote the marker-assisted breeding and international communication of this marvelous crop.

## Introduction

The germplasm identification and classification of ornamental plants is essential for their breeding, of which the former is an important basis for the latter. The combination of these two plays a decisive role in the exploration of utility potential of germplasm to the utmost extent, and also lays foundations for the genetic breeding, popularization and international communication of ornamental plants [1], [2].

Chrysanthemum (*Chrysanthemum*×*morifolium* Ramat.) is a famous traditional flower in China that possesses great aesthetic value and abundant cultural associations, making it the most economically important horticultural crop worldwide [3]. Chinese traditional chrysanthemums are the most popular potted, cut and garden ornamentals with a market share of 21% in China [4], with a comparable market share in other Asian countries. The success of Chinese traditional chrysanthemums is based on the abundant diversity of flower type, color and plant architecture [5]. Such a rich diversity coincides with the genomic complexity of cultivated chrysanthemums worldwide, which is part of an allohexaploid hybrid complex [6]. Cultivated chrysanthemums are generally thought to be a result of natural hybridizations among species of *Chrysanthemum sinense*, *C. erubescens*, *C. ornatum*, *C. japonense*, *C. makinoi*, *C. chanetii*, *C. vestitum*, *C. indicum*, *C. lavandulifolium* and *C. zawadskii* [7], [8]. As an out-breeding and self-incompatible ornamental crop, cultivated chrysanthemums are highly heterozygous [9]. Thus, Chinese traditional chrysanthemums are complex hybrids that contain genetic material from multiple species, which were utilized over hundreds of years of chrysanthemum breeding. The identification and classification of the current breeding materials would be helpful to better utilize the germplasm and provide references for innovations in plant improvement and production systems of chrysanthemum.

During the long period (more than 1600 years) of natural and artificial selection of Chinese traditional chrysanthemum cultivars, high similarity in phenotypic characteristics due to the influence of environmental conditions and cultivation methods as well as duplicated hybridization among old and modern cultivars has gradually emerged among many cultivated chrysanthemums, resulting in a serious phenomenon of homonyms and synonyms within this important crop [10]. This problem has restricted the accurate identification of chrysanthemums to a large extent, and has hindered plant breeding, cultivar protection and registration, and international communication. Unfortunately, effective solutions to this problem by exploiting either morphological or molecular aspects have not been proposed.

Japan, USA, UK and China are four important countries that have focused on the study of chrysanthemum classification, and accordingly, several classification systems for chrysanthemums have been proposed [1], [11], [12], [13], [14]. Both the Japanese and American classification criteria involve flower diameter and the flower head type. However, the flower diameter is considered to be the first criterion in America [15], which is consistent with most of the Chinese systems; while in Japan, the flower diameter is used as the second criterion [16]. The classification system is very simple in England, where flower head type is the only classification criterion [17]. However, these classification systems are not comprehensive enough to classify Chinese traditional chrysanthemums, resulting in many controversies regarding the classification of cultivars with either similar or obviously different morphological features [10]. In contrast, although the Chinese classification system for chrysanthemum is significantly more comprehensive and can be used on more cultivars [1], its scientific foundation needs to be approved by biological research data.

The identification and classification of ornamental plants can be evaluated using molecular markers, due to their large number in a typical plant genome and excellent stability compared with the morphological markers. Especially, cultivars possess similar morphological features can be distinguished by molecular markers very well. In recent years, random amplified polymorphic DNA (RAPD), amplified fragment length polymorphism (AFLP), inter-simple sequence repeats (ISSR), simple sequence repeats (SSR) and sequence-related amplified polymorphism (SRAP) markers have been used to identify sports or classify cultivars in chrysanthemum [14], [18], [19], [20], [21], [22], [23], [24], [25], [26], [27]. Among these markers, SSR markers have gained considerable popularity due to their many desirable attributes, including hypervariability, a multiallelic nature, codominant inheritance, reproducibility, relative abundance, extensive genomic coverage (including organellar genomes), chromosome-specific location, amenability to automation, and high-throughput genotyping [28]. However, the application of SSR markers in the identification and classification studies on chrysanthemums is scarce.

The objective of this study was to identify a collection of Chinese traditional chrysanthemum cultivars that are currently available in Asian markets using SSR fingerprints and molecular identities, and to further testify the accuracy and rationality of the Chinese classification system for chrysanthemums [1], [29] using clustering, population structure and principal component analyses (PCA). Moreover, because the Q-matrices (the probability of each cultivar be clustered into each subpopulation) were obtained as a covariant factor, the goal was to further identify the first associations between genomic SSR markers and horticulturally important traits, thereby avoiding the effect of linkage disequilibrium on the accuracy of the association analysis to a large extent, which may serve as auxiliary evidences for the classification and starting points for further detailed analyses of the genetic architecture of these important traits to promote the marker-assisted breeding of this marvelous crop.

## Materials and Methods

### Plant materials

A collection of 480 Chinese traditional chrysanthemum cultivars was obtained from the Chrysanthemum Nursery of Beijing Forestry University, Beijing, China (Table S1). These genotypes were chosen because they are representative samples of the gene pool that is currently used in China, which covers all of the five petal types, thirty flower head types and nine flower color groups that have been described in the Chinese traditional classification system of chrysanthemum cultivars [1], [29]. Conventional field management and operating processes for Chinese traditional chrysanthemum cultivars were adopted: robust cutting slips with 7–8 cm in length were cut from mother plants in April, 2008–2012, and subsequently placed into plugs (turf: vermiculite = 3∶1, v/v). Seedlings with strong roots were then transferred into separated pots with the sizes of 10×10 cm after 25 d, and then placed on furrows in the shed. During this period of growth, watering plants and fertilizing them with 0.1% (w/v) nitrogenous fertilizer every other week. During the entire period of growth, the daily mean temperature, mean humidity and pH value of soil were 20±2°C, 65% and 6.5, respectively. When the stems grew to 25 cm in length, the photoperiod and minimum air temperature were 13.5 h and 15±2°C, respectively.

### Phenotyping

A continuous five-year distinctness-uniformity-stability test (2008–2012) for 48 phenotypic traits of the selected cultivars was performed according to the Ministry of Agriculture of the People's Republic of China [30], including six stem traits, 21 flower traits and 21 leaf traits. The stability of these traits had been verified previously [31]. The plants were cut back at the end of each year and measurements were taken. Ten plants from each cultivar were measured. The numerical values of the quantitative traits were the mean value of five years of data, while the descriptions of the qualitative polymorphic traits were the mode values (the most frequently occurred values), which were evaluated using numbers to convert them into quantitative values. These traits are summarized in Table S1 and S2.

### SSR analysis

Fresh, young leaf samples of each cultivar were collected from a chrysanthemum conservancy in Beijing. The total genomic DNA of each cultivar was extracted from the fresh leaves using the Extractive Kit for Plant Genomic DNA (Tiangen Biotech Co., Beijing, China).

A total of 20 polymorphic SSR loci from chrysanthemums that had been previously verified using 40 cultivars with known genetic relationships [14], and showed high polymorphisms were selected as loci in the present study. The PCR reaction system was based on Li et al [32] with several important modifications. Briefly, 100 ng of DNA template, 2.0 mmol·L^−1^ Mg^2+^, 0.1 mmol·L^−1^ dNTP, 0.5 µmol·L^−1^ each of forward and reverse primer, and 1 U *Taq* DNA polymerase were mixed in a 20-µL reaction system. All reactions were performed using a TProfessional Standard Gradient Thermocycler (Biometra GmbH, Goettingen, Germany). The following thermal cycling protocol was used: an initial melting at 94°C for 5 min followed by 35 cycles of 94°C for 50 s, 45 s at the optimum annealing temperature [14], and 72°C for 50 s. After a final extension step of 72°C for 10 min, the reaction mixture was held at 4°C. Amplified PCR products were separated using capillary electrophoresis according to Grossman and Colburn [33].

The original data, with an *fsa* file format, were obtained after analyzing the amplification products using GeneMarker software (version 1.85; SoftGenetics, State College, PA, USA); next, an original matrix composed of “0” and “1” was obtained and formatted as an *xls* file, in which “0” represents the absence of amplified fragments and “1” represents the presence of amplified fragments.

### Establishment of DNA fingerprints and molecular identities for cultivar identification

Only different cultivars in nature can be used for clustering, population structure and marker-trait association analyses. Therefore, to investigate whether the selected cultivars are separate and unique individuals or not, we first identified 400 random cultivars using the unique DNA fingerprints and molecular identities established by the core loci. The other 80 cultivars were used to verify the validity of the molecular identities we established (Table S1). The detailed methods are described in below.

Firstly, eleven parameters related to polymorphism and discriminability of selected loci were analyzed using Popgene (version 1.32, University of Oulu, Oulu, Finland) and Microsoft Excel 2010 (Microsoft, Redmond, WA, USA) software; the parameters included the observed number of bands (NB), the observed number of alleles (NA), the mean value of effective alleles (NE), the number of polymorphic alleles (NPA), the proportion of polymorphic alleles (PPA), the number of specific alleles (NSA), the proportion of specific alleles (PSA), the polymorphism information content (PIC), the mean Shannon's information index (I), the mean Nei's gene diversity index (H), and the discriminating power (DP).

Secondly, the Technique for Order Preference by Similarity to an Ideal Solution (TOPSIS) method [34], [35] was used to evaluate the comprehensive polymorphism and discriminability of all loci. The plus and minus ideal solutions of all parameters were calculated, and the distances between the parameters and the plus ideal solutions, the minus ideal solutions, and the relative approach degrees of different loci were calculated using Microsoft Excel 2010 software. According to the rank of the loci, the core loci used for the establishment of unique SSR fingerprints and molecular identities were screened out using single locus or multiple loci, until all cultivars were distinguished.

Finally, the DNA fingerprints of each cultivar were established using Microsoft Excel 2010 software, which was then transformed to barcodes (molecular identities) that comprised several binary digits, according to the rule that “0” represents the absence of an amplified band and “1” represents the presence of an amplified band. After that, to keep the digits of each molecular identity within a minimum level, the binary identities were further transformed to denary ones.

### Clustering analysis

Using the NTSYS (version 2.1; Applied Biostatistics, Port Jefferson, NY, USA) software, a depiction of the Q-mode clustering analysis of the selected cultivars based on the polymorphic alleles was constructed using the Unweighted Pair-group Method with Arithmetic Means based on Nei's genetic distances among different cultivars [36].

### Population structure and PCA

To infer population structure and to assign individuals to distinct subpopulations, we further determined the genetic distances and population structure using a model-based method. The population structure was calculated using STRUCTURE software (version 2.3.4; Stanford University, CA, USA) [37], [38], [39], [40] with a burn-in of 10,000 followed by 100,000 iterations. We selected the implemented ‘admixture model’ and conducted 20 independent runs for each K to reduce random errors. We tested subpopulation numbers (K) between 2 and 14. To estimate the appropriate value for K, we used two approaches: (1) from the maximum lnP(D) value with high variance among different runs as previously described by Rosenberg et al. [41] and (2) use of an AdHoc method based on the second order rate of change of the likelihood (ΔK) as previously described by Evanno et al. [42]. Calculations and graph construction were performed in Microsoft Excel 2010.

To investigate the accuracy of the classification results, the PCA was further performed with allele frequencies calculated from the polymorphic alleles using the Multivariate Statistical Package version 3.13b (Kovach Computing Services, Anglesey, Wales, UK) [43].

### Association analysis

Putative associations between the polymorphic SSR markers and phenotypic traits were calculated using the Q-matrix values of the estimated appropriate K value as a cofactor [44]. Associations were calculated using TASSEL software (version 5.0; http://www.maizegenetics.net). We used the implemented MLM approach and 1,000 permutations. The significance threshold was Bonferroni-adjusted and -lg-transformed. A marker was considered associated if its -lg *P*-value was larger than 2.00 (*P*<0.01) [31].

## Results

### Establishment of DNA fingerprints and molecular identities for cultivar identification

Marker data were generated from 20 SSR loci with a total of 43,091 bands and 210 alleles with mean values of 2154.55 and 10.50, respectively (Table S3). Among the 210 alleles, 204 (97.14%) were polymorphic. The maximum and minimum values of NB occurred in JH20 (4455) and JH30 (855), respectively, while for NA, they occurred in JH27 (22) and JH03 (2), respectively. The maximum, minimum, total and mean values of NE were 9.28 (JH20), 1.78 (JH03), 89.78 and 4.49, respectively. The PPA of most of the loci reached 100% except for JH13 (60%), JH15 (75%), JH32 (85.71%) and JH48 (88.89%). JH27 possessed the highest NSA value (11), and the total and mean values of this parameter were 54 and 2.70, respectively. The discrepancy of PSA differed greatly among the selected loci, which varied from 0 to 59.09% (JH27), and the mean value of this parameter was 20.25%. For PIC, I and H, the maximum values were 0.94 (JH20), 0.65 (JH03) and 0.67 (JH32), respectively, while the minimum values were 0.50 (JH03), 0.23 (JH27) and 0.26 (JH28), respectively. The DP values of JH31 (384), JH48 (369), JH18 (352), JH32 (337), JH10 (336), JH12 (321) and JH33 (311) were larger than 300, indicating a high discriminating power for chrysanthemum cultivar identification of these loci. On the contrary, the discriminating power of JH03 was the poorest, which could identify only four cultivars (Table 1). The above mentioned results indicated that although most of the selected loci were informative, JH03 was not suitable for the further establishment of DNA fingerprint and molecular identity; while the 11 perimeters that were related to the polymorphism and discriminability varied greatly among the other 19 loci, making confusions to justify whose polymorphisms were relatively higher. Therefore, a comprehensive evaluation system for the polymorphism and discriminability of the selected loci needs to be further established.

**Table 1:** Characteristics of twenty SSR loci used in the present study detected by Popgene software.

Locus	NB	NA	NE	NPA	PPA%	NSA	PSA%	PIC	I	H	DP
JH03	856	2	1.78	2	100.00	0	0	0.50	0.65	0.29	4
JH04	3,839	15	8.00	15	100.00	2	13.33	0.92	0.34	0.32	247
JH08	2,388	7	4.98	7	100.00	1	14.29	0.83	0.47	0.31	188
JH09	1,738	8	3.62	8	100.00	2	25.00	0.82	0.46	0.34	239
JH10	2,293	15	4.78	15	100.00	3	20.00	0.87	0.31	0.41	336
JH11	2,372	8	4.94	8	100.00	0	0	0.85	0.47	0.37	152
JH12	1,086	10	2.26	9	90.00	4	40.00	0.77	0.38	0.48	321
JH13	1,639	5	3.41	3	60.00	0	0	0.75	0.48	0.37	162
JH15	1,665	4	3.47	3	75.00	0	0	0.74	0.56	0.34	174
JH18	1,074	4	2.24	4	100.00	0	0	0.66	0.54	0.64	352
JH20	4,455	18	9.28	18	100.00	1	5.56	0.94	0.31	0.37	185
JH27	1,631	22	3.40	22	100.00	13	59.09	0.85	0.23	0.42	261
JH28	2,888	15	6.02	15	100.00	3	20.00	0.91	0.34	0.26	255
JH30	855	12	1.78	12	100.00	6	50.00	0.68	0.32	0.44	276
JH31	2,145	10	4.47	10	100.00	2	20.00	0.82	0.38	0.62	384
JH32	1,831	7	3.81	6	85.71	3	42.86	0.77	0.41	0.67	337
JH33	4,194	19	8.74	19	100.00	8	42.11	0.91	0.27	0.52	311
JH47	1,275	12	2.66	12	100.00	5	41.67	0.78	0.34	0.48	233
JH48	3,125	9	6.51	8	88.89	1	11.11	0.86	0.37	0.66	369
JH75	1,742	8	3.63	8	100.00	0	0	0.86	0.51	0.41	137
Total	43,091	210	89.78	204	—	54	—	—	—	—	—
Mean value	2,154.55	10.50	4.49	10.20	97.14	2.70	20.25	0.80	0.41	0.44	246.15

NB, NA, NE, NPA, PPA, NSA, PSA, PIC, I, H and DP represent the observed number of bands, the observed number of alleles, the mean value of effective alleles, the number of polymorphic alleles, the proportion of polymorphic alleles (%), the number of specific alleles, the proportion of specific alleles (%), the polymorphism information content, the mean Shannon's information index, the mean Nei's gene diversity index, and the discriminating power, respectively.

Using the TOPSIS method, the standard dimensionless matrix for the 11 parameters of the 20 loci was constructed based on the data shown in Table 1. Afterward, a comprehensive evaluation system for the polymorphism and the discriminability of the selected loci was successfully established (Table 2). A total of 63 alleles, with amplicon sizes ranging from 105 bp to 389 bp, were amplified by the five best-scoring loci (JH31, JH48, JH18, JH32 and JH13), which distinguished all samples.

**Table 2:** The standard dimensionless matrix of the eleven parameters related to the polymorphism and discriminability of the 20 SSR loci evaluated by the TOPSIS method.

Locus	NB	NA	NE	NPA	PPA	NSA	PSA	PIC	I	H	DP	Dj^+^	Dj^−^	Zi	Rank
JH03	0.10	0.10	0.10	0.10	0.07	0.00	0.00	0.46	0.33	0.30	0.23	0.0646	0.1147	0.4525	20
JH04	0.27	0.14	0.27	0.14	0.26	0.11	0.26	0.37	0.22	0.31	0.27	0.0329	0.0812	0.5003	15
JH08	0.30	0.17	0.30	0.17	0.48	0.10	0.25	0.48	0.35	0.22	0.24	0.0371	0.0556	0.6002	9
JH09	0.26	0.21	0.26	0.21	0.31	0.09	0.13	0.34	0.32	0.27	0.21	0.0217	0.0438	0.4809	18
JH10	0.31	0.28	0.31	0.28	0.29	0.21	0.24	0.38	0.25	0.25	0.36	0.0164	0.0583	0.5014	14
JH11	0.28	0.20	0.28	0.20	0.14	0.00	0.00	0.33	0.29	0.22	0.23	0.0233	0.0428	0.6397	6
JH12	0.38	0.09	0.28	0.09	0.24	0.18	0.13	0.34	0.28	0.32	0.38	0.0277	0.0485	0.4977	16
JH13	0.25	0.15	0.25	0.15	0.53	0.00	0.00	0.50	0.41	0.29	0.22	0.0378	0.0704	0.6506	5
JH15	0.26	0.11	0.26	0.10	0.45	0.05	0.21	0.49	0.48	0.26	0.23	0.0355	0.0549	0.6076	8
JH18	0.09	0.08	0.09	0.08	0.45	0.00	0.00	0.44	0.45	0.43	0.42	0.0350	0.0701	0.6665	3
JH20	0.22	0.10	0.22	0.10	0.42	0.08	0.28	0.33	0.22	0.38	0.26	0.0271	0.0532	0.4825	17
JH27	0.16	0.41	0.16	0.41	0.41	0.25	0.23	0.40	0.14	0.26	0.28	0.0374	0.0474	0.5591	12
JH28	0.22	0.06	0.15	0.09	0.21	0.34	0.25	0.40	0.20	0.28	0.29	0.0432	0.0509	0.5349	13
JH30	0.12	0.32	0.12	0.32	0.50	0.10	0.14	0.45	0.23	0.33	0.36	0.0377	0.0527	0.5833	10
JH31	0.25	0.20	0.25	0.20	0.42	0.08	0.16	0.41	0.32	0.39	0.42	0.0255	0.0802	0.7583	1
JH32	0.23	0.16	0.23	0.14	0.38	0.09	0.23	0.43	0.31	0.45	0.39	0.0378	0.0714	0.6534	4
JH33	0.33	0.31	0.33	0.31	0.33	0.33	0.33	0.31	0.14	0.26	0.27	0.0398	0.0672	0.6279	7
JH47	0.11	0.29	0.11	0.29	0.43	0.30	0.42	0.40	0.19	0.31	0.26	0.0375	0.0505	0.5737	11
JH48	0.31	0.19	0.31	0.18	0.37	0.08	0.15	0.40	0.34	0.39	0.38	0.0339	0.0688	0.6701	2
JH75	0.23	0.22	0.23	0.22	0.16	0.00	0.00	0.38	0.32	0.29	0.22	0.0196	0.0528	0.4726	19

NB, NA, NE, NPA, PPA, NSA, PSA, PIC, I, H and DP represent the observed number of bands, the observed number of alleles, the mean value of effective alleles, the number of polymorphic alleles, the proportion of polymorphic alleles (%), the number of specific alleles, the proportion of specific alleles (%), the polymorphism information content, the mean Shannon's information index, the mean Nei's gene diversity index, and the discriminating power, respectively. Dj^+^, Dj^−^ and Zi represent the distance between the Pi to the plus ideal solution, the distance between the Pi to the minus ideal solution, and the relative approach degree, respectively, which were calculated according to Boran et al [34] and Torfi et al [35].

**Figure 1:**
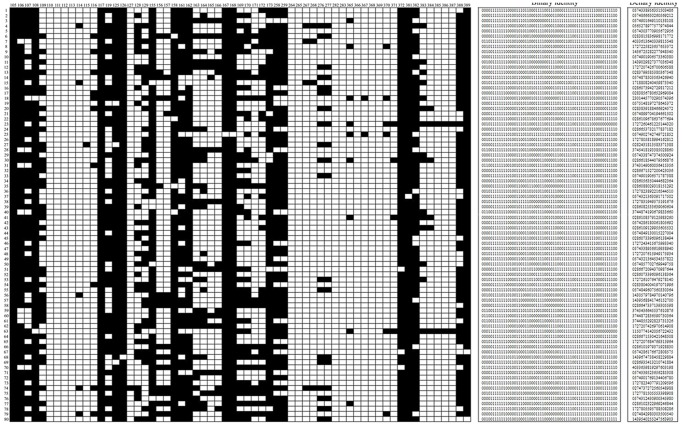
Part of the SSR fingerprints and the corresponding molecular identities of 480 Chinese traditional chrysanthemum cultivars established by five core loci. For the SSR fingerprints, the white blocks represent the presence of amplified fragments, which were transformed to “1” in the binary identities; the abscissa represents the amplified 63 alleles that ranged from 105 to 389 bp, which were reordered according to the fragment lengths of the top five loci evaluated by the TOPSIS method; the vertical axis represents the sample code of the selected cultivars. The denary identities were transformed from the binary identities.

From small to large, the fragment lengths of the 63 alleles were reordered. Accordingly, unique fingerprints and binary molecular identities with 63 digits for each cultivar were established. After transformation, the denary molecular identities with 19 digits were obtained (Table S1, Figure 1). Finally, the other 80 cultivars were amplified using the selected loci, and their molecular identities were established using the same methods mentioned above. The results showed that all of the 80 cultivars could be distinguished by their molecular identities, which showed no repetitions compared with the 400 cultivars used in the library set. Therefore, the 480 Chinese traditional chrysanthemum cultivars we selected were separate individuals in nature, all of which could be used in the further clustering, population structure and marker-association analyses.

### Clustering analysis based on Nei's genetic distances

**Figure 2:**
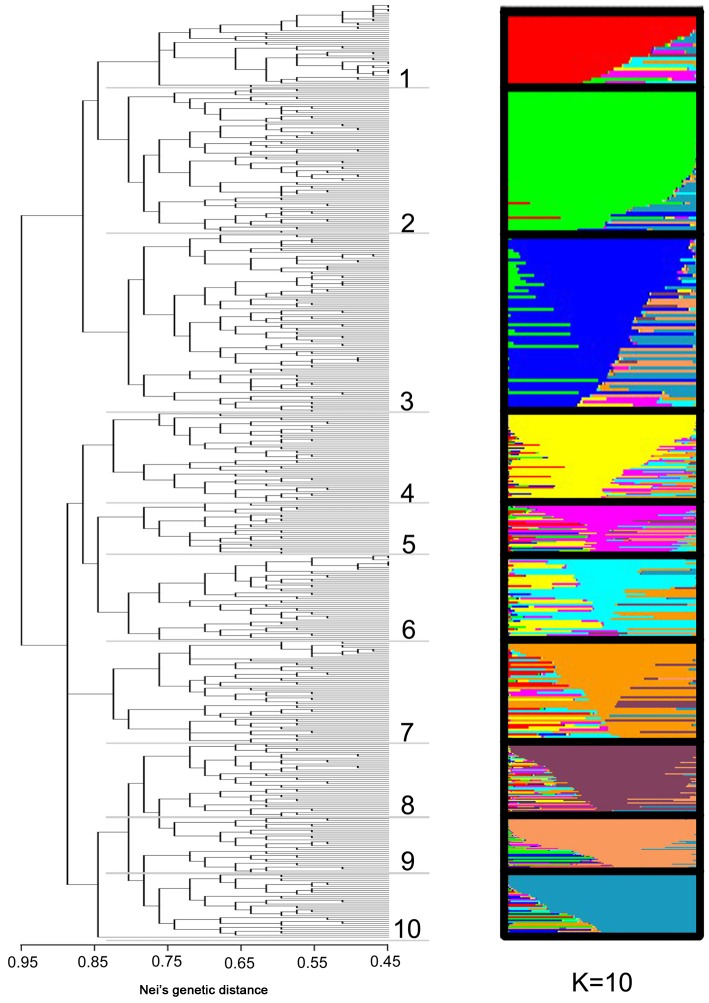
Comparison between the cluster analysis based on Nei's genetic distances and the appropriate subpopulation number (K = 10) detected using STRUCTURE 2.3.4. Each gray scale represents one subpopulation and was assigned separately for the estimated appropriate value for K = 10. As many as ten groups were clustered at the Nei's genetic distance of 0.836. Group 1: brown and dark-red cultivars; Group 2 and 3: cultivars with tubular petal type; Group 4 and 5: modern cultivars; Group 6: old cultivars; Group 7: cultivars with anemone petal type; Group 8: red cultivars; Group 9: orange cultivars; Group 10: pink cultivars.

First, information on the genetic relationships among the selected 480 Chinese traditional chrysanthemum cultivars was obtained from a Q-mode cluster dendrogram based on Nei's genetic distances, which was computed from the 204 polymorphic alleles (Figure 2). In this dendrogram, the selected cultivars clustered into ten groups at the Nei's genetic distance of 0.836, which all in all reflected the horticultural classification of chrysanthemums.

The first group mainly contained the brown and dark-red cultivars. The proportion of these two types to all of the cultivars in this group was 92.31%. In the second and third group, cultivars that demonstrated tubular ray florets (83.33%) were found, but with a mixture of small quantities of flat and spoon types. Group 4 and 5 represented so-called ‘modern cultivars’ which originated from wide-ranging cultivar hybridization after the second half of the twentieth century. Cultivars located in these two groups showed rich phenotypic variations, where group 4 consisted of mainly irregular ray florets (79.24% of which exhibited a fluttered form of the flower head type) and group 5 consisted of appurtenances on the ray florets (75.86% of which exhibited a chenille-like form and dragon-claw-like form of the flower head type). Group 6 represented the so-called ‘old cultivars,’ which were mostly bred prior to the second half of the twentieth century. These cultivars have regular ray florets, of which the cultivars with flat and spoon petal types occupied a large proportion (85.21%). In contrast to cultivars of groups 4 and 5, the flower colors of this group were mostly (74.29%) white and yellow. The cultivars of group 7 showed flowers that were shaped like anemones with well-developed tubular florets and various forms of ray florets. Finally, cultivars with red (81.57%), orange (94.38%) and pink (95.43%) ray florets were found in groups 8, 9 and 10, respectively (Figure 2).

Although the clusters corresponded very well to the horticultural classification, specific differences were observed relative to the Chinese traditional classification system of chrysanthemums. For example, cultivars that have flat, spoon and tubular ray florets could not be distinguished very clearly; and also cultivars with most of the flower head types and flower colors mixed together. Moreover, the values of Nei's gene diversity within each of the ten clusters were quite close to the diversity of the entire dataset (Table 3), indicating that the differences of gene diversity among these ten clusters were not significant. Comparatively speaking, however, the gene diversities of red (group 8, 0.374) and modern cultivars (group 5, 0.311) were relatively higher.

**Table 3:** Analysis of Nei's gene diversity in subdivided populations according to Nei [45].

Population sampled	Cultivar number	Gene diversity	SD
Cluster 1	26	0.257	0.195
Cluster 2	90	0.266	0.196
Cluster 3	83	0.269	0.189
Cluster 4	53	0.284	0.197
Cluster 5	29	0.311	0.164
Cluster 6	47	0.268	0.197
Cluster 7	50	0.195	0.207
Cluster 8	34	0.374	0.188
Cluster 9	36	0.259	0.183
Cluster 10	32	0.243	0.162
All cultivars	480	0.398	0.159

The measurements were computed for each cluster separately and among all of the cultivars examined.

### Population structure analysis and PCA

**Figure 3:**
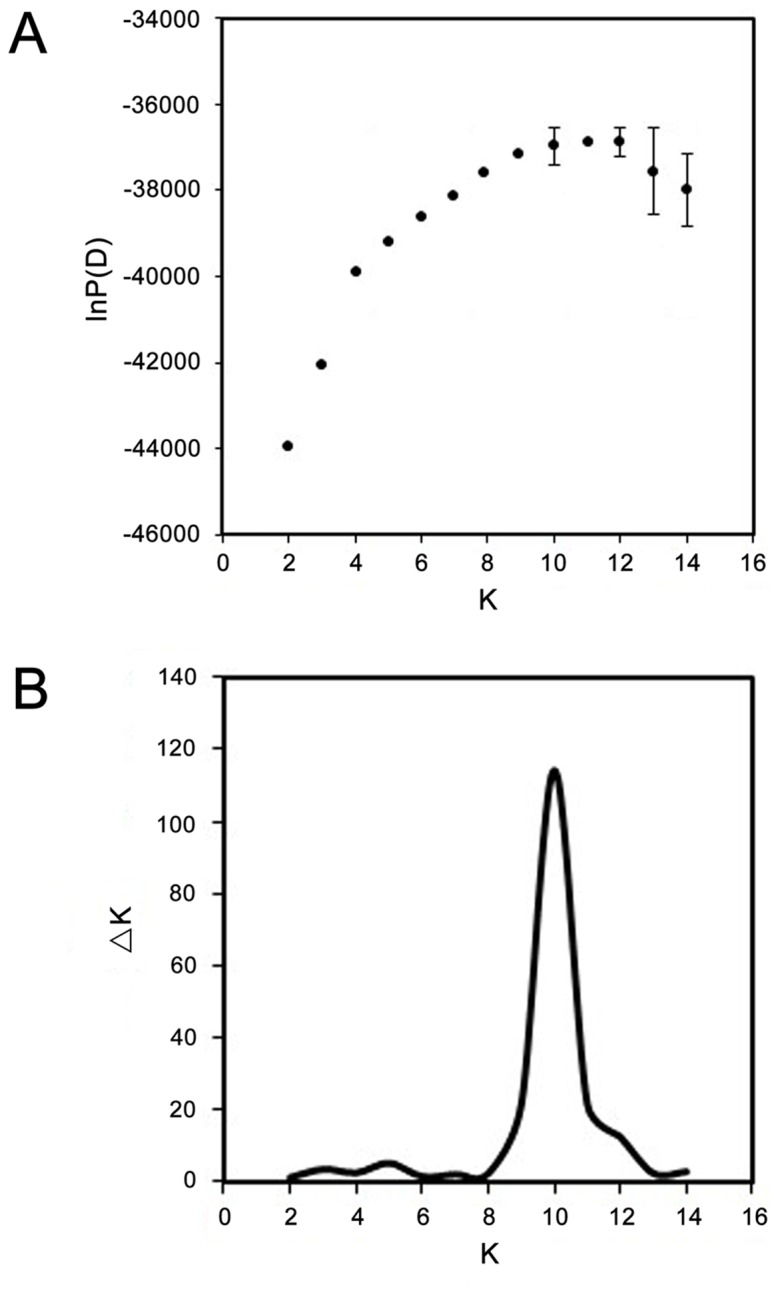
Estimation for the best subpopulation numbers based on the appropriate K values. (A) lnP(D) values revealed using STRUCTURE 2.3.4 by 20 separate runs with K values between 2 to 14. The separate 20 runs of the software revealed 10, 11 or 12 subpopulations, which showed a stable increase with small variations. (B) The mean ΔK values of 20 separate runs with K values between 2 to 14 based on lnP(D) values. The mean values of ΔK among the 20 runs reached a maximum at K = 10.

To testify the accuracy of the classification results obtained from the Nei's genetic distances, population structure analysis and PCA were performed separately. A complete set of graphs for an assumed number of subpopulations ranging from K = 2 to K = 14 is provided in Figure S1. The method described by Rosenberg et al. [41] revealed 10, 11 or 12 subpopulations among the separate 20 runs of the software, which showed a stable increase with small variations (Figure 3A). Although we could not determine the exact number of subpopulations unambiguously, we narrowed the number of subpopulations down to between 10 and 12 using this method. However, the AdHoc method of Evanno et al. [42] showed that the mean values of ΔK among the 20 runs reached a maximum at K = 10, which was much higher than the values at K = 11 and 12 (Figure 3B). Thus, after comprehensively analyzing the results of these two methods, K = 10 (ten subpopulations) was considered to be the most appropriate value.

We then compared the Q-mode cluster dendrogram based on Nei's genetic distances with the population structure using K = 10, as revealed using STRUCTURE 2.3.4. The two were consistent, and the major clusters of the cluster dendrogram corresponded to the subpopulation groups as computed using STRUCTURE 2.3.4 (Figure 2).

**Figure 4:**
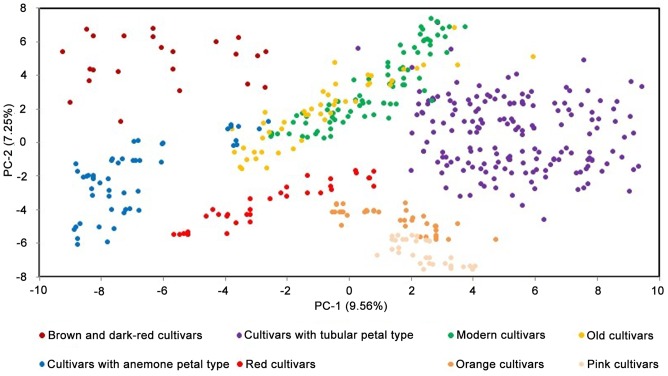
The first two axes of a principal component analysis representing the microsatellite data obtained from 480 Chinese traditional chrysanthemum cultivars. The brown and dark-red, red, orange, pink, tubular, anemone, modern and old cultivars were selected as phenotypic indicators to justify the space distributions of each group, which were determined from the cluster analysis based on Nei's genetic distances of the 204 polymorphic alleles.

The results of the clustering and population structure analyses were also in agreement with the PCA results to a large extent (Figure 4), which showed that the brown and dark-red cultivars, the cultivars with tubular and anemone petal types, the red, orange and pink cultivars could be separated clearly among each other. However, groups between ‘modern cultivar’ and ‘old cultivar’ mingled together.

### Trait-marker associations

Based on the 204 polymorphic SSR marker fragments and the statistical approaches described above, we identified 42 markers that were associated with 19 phenotypic traits (Table S4). The MLM approach using TASSEL software revealed one marker for the height of the stem, angle of the outer-layer ray florets, diameter of the disc florets, number of the disc florets and width of the leaf; two markers for the width of the stem, length of the internode, length of the ray florets, bending of the outer-layer ray florets, bending of the inner-layer ray florets, length of the cephalophorum, petal type and length of the leaf; three markers for the diameter of the capitulum, tip shape of the ray florets, length of the disc florets and flower head type; four markers for the width of the ray florets and five markers for the flower color. Markers associated with flower traits were greater than those associated with the stem and leaf traits, and some of these markers showed a significant *P*-value for more than one trait, for example, JH09_264, JH28_346, JH28_360, JH47_389, JH47_391, and JH48_383 (Table S4).

## Discussion

### The advantages and limitations of SSRs used for the identification and classification of chrysanthemums

The Chinese traditional chrysanthemum cultivars had an admixture of multifarious polyploidy ranging from pentaploid to octoploid [46], and the ploidies of most of the cultivars we selected were unknown, which resulted in difficulties and disturbances in analyzing the SSR data. Moreover, suitable mathematical models for the data analysis of codominant markers in chrysanthemums had not been developed yet. Thus, the SSRs were used as dominant markers in the present study, and the advantage of its codominant-inheritance characteristic was buried. Nevertheless, many other advanced attributes of SSR markers can be utilized in the identification and classification studies in chrysanthemums, such as hypervariability, multiallelic nature, reproducibility, relative abundance and high-throughput genotyping [28], most of which are deficient in many other markers. Expectedly, we obtained 210 alleles in the present study, whose polymorphism reached 97.14%. Thus, although the codominant-inheritance characteristic of SSR markers might be buried, it was still considered an ideal approach for the identification and classification of chrysanthemums [14].

### SSR fingerprinting and molecular identity for the identification and classification of field crops and ornamental plants

In recent years, SSR fingerprinting has been demonstrated to be a powerful tool for plant cultivar classification and identification. In the present study, the selected loci were evaluated according to their relative approach degrees using the TOPSIS method, and their ranks were reordered. According to the result, the core loci used for the establishment of unique SSR fingerprints and molecular identities were screened out using, for instance, single primers, combinations of two primers, or combinations of three primers, until all cultivars were distinguished. This comprehensive evaluation system measured these parameters scientifically and objectively, avoiding the subjectivity and fuzziness of natural-language-based description. Thus, the evaluation result was more accurate and reliable. On this basis, the 480 Chinese traditional chrysanthemum cultivars we selected were identified as separate individuals in nature, many of which were difficult to be identified using the morphological markers [10], indicating high effectiveness and reliability of the SSR markers for chrysanthemum identification.

### Classification analysis based on Nei's genetic distances

We calculated the genetic relationships of a collection of Chinese traditional chrysanthemum cultivars on the basis of 204 polymorphic SSR markers. The cluster dendrogram based on Nei's genetic distances revealed clusters of cultivars that were mostly consistent with the horticultural classification of chrysanthemums, which is based on morphological markers. We had previously selected 16 morphological markers of 735 Chinese traditional chrysanthemum cultivars to perform diversity and relativity analysis using seven multivariate statistical analysis methods, and all of the cultivars were classified into four groups and 18 forms by cluster and discriminant analysis, which mainly clustered according to their petal types and flower head types [10]. In the present study, cultivars of tubular, peculiar and anemone types could be distinguished very well in groups 2, 5 and 7, respectively, indicating the stable heredity of phenotypic traits related to ray florets of chrysanthemum. In another study, Zhang et al. [46] classified 40 typical Chinese traditional chrysanthemum cultivars into five petal types as flat, spoon, tubular, anemone and peculiar using eight karyotype parameters, and they also found that some karyotype parameters were closely related to the phenotypic traits, which was partially consistent with our study. Thus, it appeared that a strong relativity existed among the morphological markers, cytological markers and molecular markers in Chinese traditional chrysanthemums. Such relativity was also found in many other cultivated crops. For example, using 492 AFLP markers, Gawenda et al. [47] analyzed the genetic diversity of 134 *Phalaenopsis* hybrids and found that the genotypes also clustered according to their horticultural classification, such as flower type and flower color. They also found that the old and new cultivars could be divided clearly into different subgroups, which was similar to the clustering results shown in groups 4, 5 and 6 in the present study. In a study of *Linum usitatissimum*, Uysal et al. [48] analyzed the genetic diversity of 493 individual plants using 310 ISSR markers and found that these assayed plants were largely grouped according to their plant types. In a study of *Hippophae* species, similar results were also obtained [49]. Thus, molecular marker studies with large sample sizes and abundant marker numbers can reflect the horticultural classification status of cultivated crops to different extents.

We found no clustering of cultivars from the leaf forms, which is most likely due to the more stable and strongly inherited characteristics of flower traits compared with leaf traits [31], as wells as the markers associated with flower traits were greater than the markers associated with leaf traits in the present study (Table S4).

Recently, Klie et al. [27] reported that no groupings of the accessions of 81 European chrysanthemum cultivars were found, either according to their common origin or horticultural type, or by similarities in important phenotypic characteristics. One potential reason is that the chrysanthemum originated in China and was introduced to Europe at the end of the seventeenth century [7]. Thus, more than 1600 years of breeding history resulted in a richer diversity and variation of Chinese chrysanthemums, compared to the diversity and variation of modern European chrysanthemum cultivars. Another potential reason is that we fortunately observed several markers associated with many phenotypic traits, and thus, the cluster analysis based on these markers reflected the characteristics of the phenotypic traits.

### Analysis of the population structure and PCA

In many crop species, experimental populations are likely to be structured because of common ancestors and a small number of generations, due to the time these ancestors contributed to the gene pool of the population [27]. Information regarding the population structure may also be used to estimate the genetic relationships among cultivars, and to testify the accuracy of the classification results based on the clustering of Nei's genetic distances due to the different algorithms of these two methods.

For the subpopulation number only, a range between 10 and 12 was determined as the most likely number using the method of Rosenberg et al. [41]. As a second approach, we used the method of Evanno et al. [42]. This method considered gene flow between subpopulations based on different models rather than assuming distinct isolated subpopulations. It showed that the mean values of ΔK among the 20 runs reached a maximum at K = 10. Therefore, we considered the results to indicate that ten subpopulations were more reliable than those computed using Prichard's method. The bar plot of K = 10 compared to the distance-based clustering dendrogram is shown in Figure 2. A first estimation of the population structure was possible, providing us with the opportunity to calculate the preliminary associations with different Q-matrices.

Basically, the topology of the clusters in the dendrogram on the basis of Nei's genetic distances is highly similar to the determination of groups using structure analysis. However, groups 2, 3, 4 and 5 separated into four independent subpopulations in the structure analysis, which represented two types of cultivars in the clustering analysis (groups 2 and 3 represented tubular cultivars, while groups 4 and 5 represented ‘modern cultivars’). Moreover, group 5 was highly admixed and could not be determined as a distinct subpopulation. The ‘old cultivars’ (group 6) formed an unstable subpopulation that was highly mixed with ‘modern cultivars’ (groups 4 and 5), which was also observed in the PCA, due to reduplicative hybridization among ‘old’ and ‘new’ cultivars, resulting in a highly admixed population. Similar results were also observed in the studies of *Hibiscus rosa-sinensis* [50], *Aechmea* [51] and *Platanus* species [52].

In conclusion, although some differences between the classification results we obtained and the Chinese traditional classification system of chrysanthemums were observed, the rationality of the classification for many petal types (tubular group, anemone group and peculiar group), flower head types (fluttered form, chenille-like form and dragon-claw-like form) and flower colors (brown, red, dark-red, orange and pink) was proved. The main reason for the observed differences might be that the SSR markers distribute randomly throughout the entire plant genome; thus, it is not surprising that many morphological characteristics do not correlated with these SSR markers. Another possible reason is that because the performances of morphological characters are the result of the expression of certain functional genes under the internal and external environment, the structural differences of the DNA are not always exposed [14].

### Trait-marker associations

The wealth of phenotypic variation and lack of segregating populations for conventional genetic analyses make association-based approaches an attractive alternative in the cultivated plants. In recent years, genome-wide association studies developed for humans have also found a broad application in cultivated plants, and they represent an alternative approach to species with a complex genetic background based on quantitative trait loci (QTL) analyses [44]. Association analyses currently exhibit enormous popularity in crop plant genetics due to their resolution of marker-trait. In the present study, we found preliminary associations between 42 unique markers and 19 ornamental traits using a Bonferroni-adjusted *P*-value of the MLM approach with TASSEL software. Furthermore, a trait-marker analysis revealed several markers associated with more than one trait. This result might be caused by pleiotropic effects of the linked genomic regions or may be due to statistical errors.

Using 19 SRAP markers, Li et al. [32] performed an association analysis of 18 phenotypic traits in 58 chrysanthemum cultivars. They found that there were six SRAP loci associated with five quantitative characters, among which three flower traits were associated with five loci, while one stem and one leaf traits were associated with one locus, respectively. However, because the number of markers was quite close to the number of cultivars in their study, deviation might occur in marker-trait association. Although the sample sizes used in the present study were even greater, because we analyzed a relatively small number of markers, and the linkage genetic maps of chrysanthemum have been rarely reported at present, we considered these associations to be preliminary and require confirmation with either additional markers or conventional QTL mapping in segregating populations. Therefore, unfortunately, these markers could not be located in the entire genome of chrysanthemum at present. Nevertheless, the next-generation sequencing has enabled rapid progress in the sequencing of even large crop genomes. Whether or not the chrysanthemum genome is sequenced in the near future, these technologies will enable high-throughput marker analyses in ornamentals, and therefore, more sophisticated association studies with an extremely high marker density will be possible.

## Supporting Information

Figure S1 **Comparison of subpopulations computed using STRUCTURE 2.3.4 with different values of K between 2 to 14.** Each color represents one subpopulation and was assigned separately for each K.
(TIF)

Table S1 **List of** ***Chrysanthemum*** **×** ***morifolium*** **cultivars used in the present study, their molecular identities and phenotyping data.** ^*^ represents the 80 cultivars used for validation of the establishment method of molecular identities for other 400 cultivars.
(XLSX)

Table S2 **Phenotypic traits recorded for the Chinese traditional chrysanthemum cultivars under study.**
(DOCX)

Table S3 **SSR scoring data of** ***Chrysanthemum*** **×** ***morifolium*** **cultivars used in the present study.** ^*^ represents the 80 cultivars used for validation of the establishment method of molecular identities for other 400 cultivars.
(XLSX)

Table S4 **The best –lg-transformed** ***P*** **-values and marker names for significant associations found in the present study.** Markers were considered associated if its -lg *P*-value was greater than 2.00 (*P*<0.01).
(DOCX)
