# Supplementary figures and images for: Application of Genomic SSR Locus Polymorphisms on the Identification and Classification of Chrysanthemum Cultivars in China
Source: PLoS One. 2014 Aug 22;9(8):e104856. doi: 10.1371/journal.pone.0104856 (PMC4141723; doi:10.1371/journal.pone.0104856)

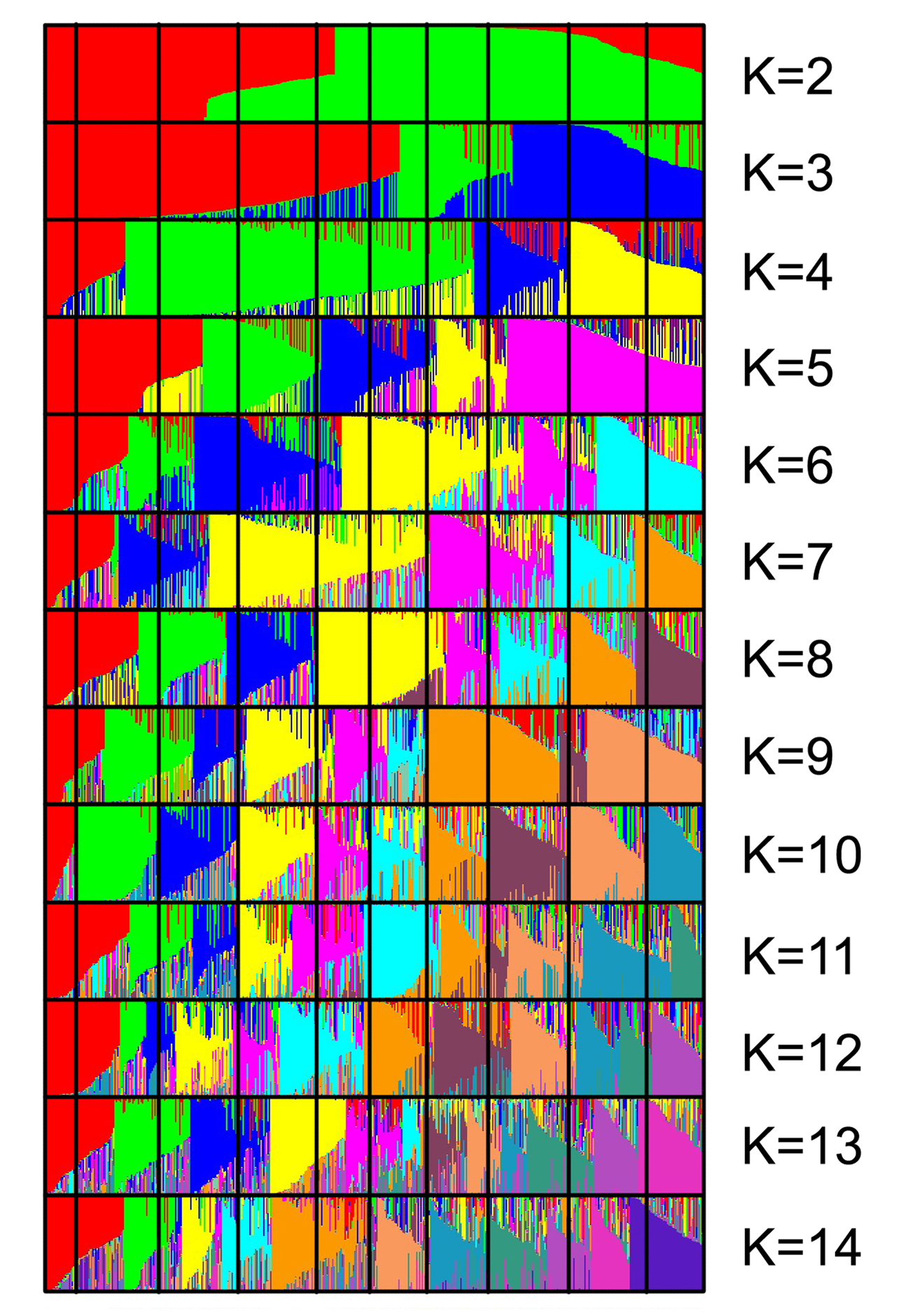

Supplement: Figure S1 — Comparison of subpopulations computed using STRUCTURE 2.3.4 with different values of K between 2 to 14. Each color represents one subpopulation and was assigned separately for each K. (TIF) [file pone.0104856.s001.tif]
