# Supplementary material for: Application of Genomic SSR Locus Polymorphisms on the Identification and Classification of Chrysanthemum Cultivars in China
Source: PLoS One. 2014 Aug 22;9(8):e104856. doi: 10.1371/journal.pone.0104856 (PMC4141723; doi:10.1371/journal.pone.0104856)
Supplement: Table S2 — Phenotypic traits recorded for the Chinese traditional chrysanthemum cultivars under study. (DOCX) [file pone.0104856.s003.docx]

**Table S2.** Phenotypic traits recorded for the Chinese traditional chrysanthemum cultivars under study.

| **Trait type** | **Trait** | **Measuring method** |
| --- | --- | --- |
| Stem trait | Height of the stem | Measure the height from the bottom of the stem to the top of the plant with a ruler (cm) |
|  | Width of the stem | Measure the width of the stem using a vernier caliper (mm) |
|  | Bending of the stem | Straight, bent |
|  | Strength of the stem | Strong, weak |
|  | Stem ridge | Obvious, inconspicuous |
|  | Length of the internode | Measure the length of the internodes with a ruler, and then calculate the mean value (cm) |
| Flower trait | Height between the bottom of the phyllary and the top of the capitulum | Detach the capitulum from the plant, and subsequently measure the height between the bottom of the bracteole and the top of the capitulum using a vernier caliper (mm) |
|  | Diameter of the capitulum | Measure the maximal transverse diameter of the capitulum with a ruler (cm) |
|  | Number of the ray florets | Detach the capitulum from the plant, and then quantify the number of the ray florets |
|  | Length of the ray florets | Detach the ray florets from the capitulum, and then measure the maximal length with a ruler (cm) |
|  | Width of the ray florets | Detach the ray florets from the capitulum, and then measure the maximal width with a ruler (cm) |
|  | Angle of the outer-layer ray florets | Antrorse, aclinic, decurrent, nodding |
|  | Bending of the outer-layer ray florets | Aclinic, inverted, incurved, screwy, antrorse, nodding |
|  | Bending of the inner-layer ray florets | Aclinic, inverted, incurved, screwy, antrorse, nodding |
|  | Tip shape of the ray florets | Cuspidal, round, jugged, bossed |
|  | Length of the disc florets | Detach the capitulum from the plant, and then measure the maximal length of the disc florets using a vernier caliper (mm) |
|  | Diameter of the disc florets | Detach the capitulum from the plant, and then measure the diameter of the disc florets using a vernier caliper (mm) |
|  | Number of the disc florets | Detach the capitulum from the plant, and then quantify the number of the disc florets |
|  | Spread of the disc florets | Without disc florets, concentrated, dispersive |
|  | Length of the peduncle | Measure the length from the top leaf to the bottom of the phyllary with a ruler (cm) |
|  | Width of the peduncle | Measure the maximal width of the phyllary with a vernier caliper (mm) |
|  | Length of the cephalophorum | Measure the length of the cephalophorum with a vernier caliper (cm) |
|  | Alabastrum shape | Flat, round, high |
|  | The position of the bracteole | Normal, mix with disc florets |
|  | Flower color | White, yellow, pink, orange, red, yellow-green, purple, brown, dark-red |
|  | Petal type | Flats, spoon, tubular, anemone, peculiar |
|  | Flower head type | Single form, lotus-like form, peony-like form, flat-pan form, stacked-spherical form, reflexed form, beehive-like form, spherical-spoon form, sparrow-tongue-like form, scattered-reflexed form, lotus-set-like form, spoon-lotus form, single-tubiform form, straight-tubiform form, tubiform-pan form, acicular form, scattered-tubiform form, tubiform-spherical form, filiform form, fluttered form, upswept-bead form, jade-like form, pendant-bead form, flat-anemone form, spoon-anemone form, tubiform-anemone form, whole-anemone form, chenille-like form, dragon-claw-like form, aristate form |
| Leaf trait | Length of the leaf | Measure the maximal length of the 10th leaf from the top leaf with a ruler (cm) |
|  | Width of the leaf | Measure the maximal width of the 10th leaf from the top leaf with a ruler (cm) |
|  | Thickness of the leaf | Measure the maximal thickness of the 10th leaf from the top leaf using a vernier caliper (mm) |
|  | Burnish of the leaf | Bright, medium, dark |
|  | Folding degree of the leaf | Flat, slightly rugate, rugate |
|  | Crimp ratio of the leaf | Strong, medium, weak |
|  | Hardness of the leaf | Hard, medium, soft |
|  | Tip shape of the leaf | Cuspidal, acute, round |
|  | Bottom shape of the leaf | Convex, flat, concave |
|  | Leaf hair | More, medium, less |
|  | Length of the petiole | Measure the maximal length of the 10th petiole from the top leaf with a ruler (cm) |
|  | Angle of the petiole | Antrorse, aclinic, decurrent |
|  | Shape of the stomium bottom of the leaf | Round, medium, sharp |
|  | First stomium of the leaf | Deep, medium, shallow |
|  | Second stomium of the leaf | Deep, medium, shallow |
|  | Overlapping of the stomium | non-lapped (wide), non-lapped (narrow), lapped |
|  | Stomium protuberance of the leaf | Yes, no |
|  | Stipule number | Yes, no, mixed |
|  | Spread of the stipule | One-side, both-sides, mixed |
|  | Stipule shape | Without stomium, double-stomiums, multiple-stomiums |
|  | Stipule size | Small, medium, large, very large |
