# Supplementary material for: Application of Genomic SSR Locus Polymorphisms on the Identification and Classification of Chrysanthemum Cultivars in China
Source: PLoS One. 2014 Aug 22;9(8):e104856. doi: 10.1371/journal.pone.0104856 (PMC4141723; doi:10.1371/journal.pone.0104856)
Supplement: Table S4 — The best –lg-transformed P -values and marker names for significant associations found in the present study. Markers were considered associated if its -lg P-value was greater than 2.00 (P<0.01). (DOCX) [file pone.0104856.s005.docx]

**Table S4.** The best –lg-transformed *P*-values and marker names for significant associations found in the present study.

| **Trait type** | **Trait** | **Number of markers associated with trait** | **Marker name** | **-lg-transformed *P*-value** |
| --- | --- | --- | --- | --- |
| Stem trait | Height of the stem | 1 | JH20_195 | 2.54 |
|  | Width of the stem | 2 | JH20_213 | 2.42 |
|  |  |  | JH12_152 | 2.10 |
|  | Length of the internode | 2 | JH48_369 | 3.52 |
|  |  |  | JH48_383 | 2.92 |
| Flower trait | Diameter of the capitulum | 3 | JH11_373 | 4.15 |
|  |  |  | JH47_389 | 3.58 |
|  |  |  | JH47_393 | 2.44 |
|  | Length of the ray florets | 2 | JH48_383 | 2.11 |
|  |  |  | JH48_385 | 2.03 |
|  | Width of the ray florets | 4 | JH33_218 | 2.60 |
|  |  |  | JH33_244 | 5.05 |
|  |  |  | JH04_326 | 3.44 |
|  |  |  | JH09_264 | 4.10 |
|  | Angle of the outer-layer ray florets | 1 | JH31_171 | 3.30 |
|  | Bending of the outer-layer ray florets | 2 | JH28_346 | 3.16 |
|  |  |  | JH28_360 | 3.13 |
|  | Bending of the inner-layer ray florets | 2 | JH28_346 | 3.23 |
|  |  |  | JH28_360 | 3.20 |
|  | Tip shape of the ray florets | 3 | JH09_260 | 2.11 |
|  |  |  | JH09_262 | 2.08 |
|  |  |  | JH09_264 | 2.10 |
|  | Length of the disc florets | 3 | JH75_165 | 3,25 |
|  |  |  | JH75_177 | 3.11 |
|  |  |  | JH47_391 | 2.70 |
|  | Diameter of the disc florets | 1 | JH32_111 | 5.40 |
|  | Number of the disc florets | 1 | JH20_201 | 6.30 |
|  | Length of the cephalophorum | 2 | JH47_371 | 5.90 |
|  |  |  | JH47_379 | 5.58 |
|  | Flower color | 5 | JH10_119 | 5.40 |
|  |  |  | JH10_135 | 5.57 |
|  |  |  | JH10_139 | 5.40 |
|  |  |  | JH08_301 | 2.10 |
|  |  |  | JH08_303 | 2.08 |
|  | Petal type | 2 | JH09_264 | 2.12 |
|  |  |  | JH47_389 | 5.33 |
|  | Flower head type | 3 | JH09_264 | 2.12 |
|  |  |  | JH47_389 | 5.96 |
|  |  |  | JH47_391 | 5.41 |
| Leaf trait | Length of the leaf | 2 | JH48_367 | 5.52 |
|  |  |  | JH47_387 | 5.70 |
|  | Width of the leaf | 1 | JH04_336 | 3.70 |

Markers were considered associated if its -lg *P*-value was greater than 2.00 (*P* < 0.01).
